# Cellular and Molecular Basis of Neurodegeneration in Parkinson Disease

**DOI:** 10.3389/fnagi.2018.00109

**Published:** 2018-04-17

**Authors:** Xian-Si Zeng, Wen-Shuo Geng, Jin-Jing Jia, Lei Chen, Peng-Peng Zhang

**Affiliations:** College of Life Sciences, Institute for Conservation and Utilization of Agro-Bioresources in Dabie Mountains, Xinyang Normal University, Xinyang, China

**Keywords:** Parkinson disease, neurodegeneration, gene mutation, neuronal death, mechanism

## Abstract

It has been 200 years since Parkinson disease (PD) was described by Dr. Parkinson in 1817. The disease is the second most common neurodegenerative disease characterized by a progressive loss of dopaminergic neurons in the substantia nigra pars compacta. Although the pathogenesis of PD is still unknown, the research findings from scientists are conducive to understand the pathological mechanisms. It is well accepted that both genetic and environmental factors contribute to the onset of PD. In this review, we summarize the mutations of main seven genes (α-synuclein, LRRK2, PINK1, Parkin, DJ-1, VPS35 and GBA1) linked to PD, discuss the potential mechanisms for the loss of dopaminergic neurons (dopamine metabolism, mitochondrial dysfunction, endoplasmic reticulum stress, impaired autophagy, and deregulation of immunity) in PD, and expect the development direction for treatment of PD.

## Introduction

Parkinson disease (PD) is the second most common neurodegenerative disease after Alzheimer disease. In 1817, an English physician, Dr. James Parkinson, firstly described the pathological characteristic of paralysis agitans (shaking palsy) (Schnabel, 2010), and the pattern was eventually called “PD” by Charcot and Vulpian in 1862. In 1912, Friederich Lewy first described the intraneuronal inclusions, namely “Lewy bodies” which define shaking palsy at a neuropathological level, and provided a detailed histopathological analysis of the nervous system to further understanding of shaking palsy. Now PD is known as a progressive, neurodegenerative disorder characterized by severe motor symptoms, including static tremor, postural imbalance, bradykinesia and muscle rigidity. α-synuclein is the main component of Lewy bodies and has a central role in PD (Spillantini et al., 1997). Epidemiological investigations have disclosed that few of PD patients are hereditable forms, while the overwhelming majority are sporadic forms (Schapira, 2008). The prevalence of PD varies with age, from 1–2% at ages of 55–65 years to 3.5% at 85–89 years(Ibanez et al., 2017). Unfortunately, the ultimate cause of PD is not well understood yet.

Although the pathological mechanisms are largely unknown, both genetic and environmental factors are considered to involve in potential pathogenesis of PD (Gan-Or et al., 2015). Polymeropoulos et al identified the first PD-linked mutation, α-synuclein (A53T), in Italian and Greek origin families in Polymeropoulos et al. (1997). Langston et al. (1983) found that four persons with notable parkinsonism after intravenous injection of an illicit drug, containing 1-methyl-4-phenyl-1,2,5,6-tetrahydropyridine (MPTP) in 1983. Afterward, many PD-linked gene mutations and several environmental factors were found. The potential mechanisms for loss of dopaminergic neurons were also well studied. In this review, we summarize the mutations of main seven genes (α-synuclein, LRRK2, PINK1, Parkin, DJ-1, VPS35 and GBA1) associated to PD and potential mechanisms for loss of dopaminergic neurons (dopamine metabolism, mitochondrial dysfunction, endoplasmic reticulum stress, impaired autophagy, and deregulation of immunity), and also expect the development direction for treatment of PD.

## Genetic Basis of PD

There is an increasing list of mutations related to the onset of PD. They account for more than 50% of early-onset cases and about 2–3% of late-onset forms of PD. Findings of gene mutations linked to the onset of familial PD cases have confirmed their critical roles in the progress of PD and made it easy to understand the molecular pathogenesis for hereditary PD.

### α-synuclein

A missense mutation in α-synuclein (A53T), encoded by the *SNCA/PARK1* gene, was firstly identified to cause a familial form of PD (Polymeropoulos et al., 1997). Soon afterward, series of missense point mutations in the N-terminal of α-synuclein, including A30P, E46K, H50Q, G51D and A53E, were also identified. *SNCA*-linked PD is often of early onset and usually progresses rapidly. α-synuclein is the main component in Lewy bodies and most is phosphorylated at Ser129 of α-synuclein, which facilitated α-synuclein fibril uptake by neurons and exacerbated the pathology progression of PD (Sato et al., 2011; Karampetsou et al., 2017). Rodriguez et al have revealed the structure of toxic core of α-synuclein as pairs of face-to-face b-sheets by using micro-electron diffraction (Rodriguez et al., 2015). The α-synuclein variants forming oligomers were more toxic than that forming fibrils, so the former triggered more severe loss of dopaminergic neurons in the substantia nigra pars compacta (SNc) in animals (Winner et al., 2011). The dosage of α-synuclein gene(SNCA) is responsible for parkinsonism (Ross et al., 2008). Point mutation and triplication carriers show almost complete penetrance, whereas penetrance in duplication carriers ranges between 30% and 50%. What’s more, SNCA triplication carriers also tend to have even earlier onset and more severe phenotype than duplication carriers (Schneider and Alcalay, 2017). Triplication of *SNCA* gene triggered unfolded protein response in induced pluripotent stem cells (iPSCs) generated from a patient with PD (Heman-Ackah et al., 2017).

Mutations of α-synuclein are toxic to dopaminergic neurons because they alter a series of intracellular signal programs. α-synuclein A53T mutation could suppress autophagy in the brain of transgenic mice at early stage and lead to synucleinopathy (Pupyshev et al., 2017). A53T also induces mitochondrial dysfunction- and endoplasmic reticulum stress-mediated cell death pathways in PC12 cells (Smith et al., 2005a). Proteasomal protein cleavage is significantly impaired by overexpression of mutant α-synuclein in dopaminergic cells, such as SH-SY5Y and PC12 (Zondler et al., 2017). Matsumoto et al. (2017) have reported that erythrocyte-derived extracellular vesicles containing α-synuclein could transmit across the blood brain barrier; this may be a new mechanism by which the brain and periphery communicate throughout the initiation and development of PD. α-synuclein specifically binds to TrkB receptors and inhibits BDNF/TrkB signaling pathway, resulting in the death of dopaminergic neurons (Fang et al., 2017; Kang et al., 2017). Another mutation A30P could promote the neurodegeneration of dopaminergic neurons via activating microglia and upregulating the expression of PHOX and Mac-1 (Zhang et al., 2007). The mutations of α-synuclein linked to PD and the pathogenesis are summarized in **Table 1**.

**Table 1:** The mutations of α-synuclein linked to PD and the Pathogenesis.

Mutation	Pathogenesis	Cell type	Reference
A53T	Inhibiting autophagy	Mice	Pupyshev et al., 2017; Zhang et al., 2017
	Decreasing expression of tyrosine hydroxylase, Bcl-2 and DJ-1	Mice	Lee et al., 2017
	Inducing mitochondrial dysfunction and activating apoptasis	SH-SY5Y	Ju et al., 2017
	Inducing mitochondrial fragmentation, mitochondrial dysfunction and oxidative stress	Rats	Bido et al., 2017
	Inhibiting autophagy	PC12	Wang K. et al., 2016
	Decreasing proteasome activity, increasing the ROS, mitochondrial cytochrome C release, endoplasmic reticulum stress, and elevating caspase-9 and caspase-12 activities	PC12	Smith et al., 2005a
	Inducing reactive oxygen species, mitochondrial depolarization, cytochrome c release, and apoptosis	PC12	Liu et al., 2011
A30P	Inhibiting autophagy	PC12	Wang K. et al., 2016
	Inducing loss of TH^+^ cells	Rats	Brito-Armas et al., 2013
	Activating microglia	Primary midbrain cells	Zhang et al., 2007
	Inducing Lewy-like dystrophic neurites and loss of dopamine neurons	Rats	Klein et al., 2002
E46K	Inhibiting macroautophagy	PC12	Yan et al., 2014
	Altering striatal neurotransmitter metabolism, inducing oxidative stress and heightening sensitivity to rotenone	Rats	Cannon et al., 2013
	Inhibiting proteasome and inducing mitochondrial depolarization	PC12	Jiang et al., 2010
	Enhancing Ser-129 phosphorylation	HEK293T, HeLa cells, and Mice	Mbefo et al., 2015
H50Q	Decreasing solubility of α-synuclein	–	Porcari et al., 2015
	Binding copper and promoting aggregation of α-synuclein	–	Rutherford et al., 2014; Mason et al., 2016; Villar-Pique et al., 2017


Both mRNA and protein levels of α-synuclein showed a double increase in PD (Farrer et al., 2004), therefore, reducing α-synuclein expression may provide a potential therapeutic approach (Lewis et al., 2008). Zharikov et al. (2015) have clarified that knockdown of α-synuclein by using a shRNA attenuates the progressive motor deficits and the degeneration of dopaminergic neurons in SNc in rotenone-induced rats model of PD. Intranasal delivery of inhibitory oligonucleotides, siRNA or antisense oligonucleotide combined with the triple monoamine reuptake inhibitor indatraline selectively decreases α-synuclein expression in the brainstem of mice and reduces levels of endogenous α-synuclein mRNA and protein in SNc (Alarcon-Aris et al., 2017). Systemic exosomal siRNA delivery also significantly reduced the aggregation of α-synuclein in SNc dopaminergic neurones of S129D α-synuclein transgenic mice (Cooper et al., 2014).

### LRRK2

Leucine-rich repeat kinase 2(LRRK2) is a multi-domain protein, containing both Rab GTPase and kinase enzymatic activities (Steger et al., 2017). LRRK2 mutations are linked with “classical” late-onset PD and accounts for 4% of hereditary PD (Ferreira and Massano, 2017). The penetrance is incomplete and age-dependent. Mutations of *LRRK2/PARK8* gene represent the highest risk of familial PD, causing autosomal dominant PD (Zimprich et al., 2004). Morbidity of PD is significantly elevating among LRRK2 G2019S mutation carriers as aging (Healy et al., 2008). LRRK2 G2019S mutation elevates mobility of α-synuclein and enhances α-synuclein accumulation in cultured primary neurons and in dopaminergic neurons in SNc of PD brain (Volpicelli-Daley et al., 2016). The mutation also promotes the transmission of Tau in the mouse brain neurons, implicating for understanding the development of tau protein neuropathology in LRRK2-linked PD (Nguyen et al., 2018). Pathogenic mutations in LRRK2 increase its kinase activities in cells and tissues, so small molecule inhibitors of LRRK2 kinase can be used to block its activity to provide neuroprotection in some PD models (West, 2017). Intracerebral injection of LRRK2 antisense oligonucleotides also decreases endogenous protein levels of normal LRRK2 and inhibits the formation of fibril-induced α-synuclein inclusions (Zhao et al., 2017).

LRRK2 not only inhibits neuronal survival but also impairs dopamine signal. LRRK2 played important roles in the death of neurons via directly phosphorylating apoptosis signal-regulating kinase 1 at Thr832 site and activating the kinase activity (Yoon et al., 2017). In model organisms, it has numerous roles within the secretory pathway and may contribute to dopaminergic signaling (Smith et al., 2005b). LRRK2 could promote neuronal death via inhibiting myocyte-specific enhancer factor 2D activity, which is required for neuronal survival (Han et al., 2017). LRRK2 G2019S mutation impairs dopamine receptor D1 internalization, leading to an alteration in signal transduction (Rassu et al., 2017). In addition, LRRK2 G2019S mutation enhances the kinase activity and results in the impairment of synaptic vesicle trafficking selectively in ventral midbrain neurons, including dopaminergic neurons (Pan et al., 2017).

LRRK2 G2019S mutation shows a close relationship with mitochondrial dysfunction. Howlett et al. (2017) have reported that this mutation induces mitochondrial DNA damage in cultured primary neurons from rat midbrain, which is dependent on LRRK2 kinase activity, and pharmacological inhibition restores the integrity of mitochondrial DNA. PD-associated LRRK2 mutations upregulate the expression of mitochondrial calcium uniporter, a mitochondrial calcium transporter and then promote the uptake of dendritic and mitochondrial calcium in cortical neurons and familial PD patient fibroblasts (Verma et al., 2017). LRRK2 G2019S binds to and enhances its interaction with mitochondrial dynamin-like protein 1(DLP1, a fission protein), which increases mitochondrial DLP1 levels and induces neuronal toxicity, mitochondrial fragmentation, and mitochondrial dysfunction (Wang et al., 2012). LRRK2 G2019S mutation also impairs mitochondrial function by decreasing the activity of respiratory chain complex IV, whereas siRNA-mediated knockdown of LRRK2 restores the mitochondrial morphology (Mortiboys et al., 2015). Ursocholanic acid and ursodeoxycholic acid improves the LRRK2 G2019-decreased mitochondrial function *in vitro* and *in vivo*, suggesting that ursocholanic acid and ursodeoxycholic acid may be promising drugs for future neuroprotective trials (Mortiboys et al., 2013, 2015). The mutations of LRRK2 linked to PD and the pathogenesis are summarized in **Table 2**.

**Table 2:** The mutations of LRRK2 linked to PD and the Pathogenesis.

Mutation	Pathogenesis	Cell type	Reference
G2019S	Enhancing α-synuclein accumulation	Mice primary neurons	Volpicelli-Daley et al., 2016
	Inducing ASK1-mediated apoptosis	PD neuronal stem cells	Yoon et al., 2017
	Inhibiting myocyte-specific enhancer factor 2D activity	Mice	Han et al., 2017
	Inducing mitochondrial DNA damage	Primary neurons from rat midbrain	Howlett et al., 2017
	Inducing mitochondrial calcium dysregulation	Primary mouse cortical neurons	Verma et al., 2017
	Increasing mitochondrial DLP1, inducing mitochondrial fragmentation, and mitochondrial dysfunction	SH-SY5Y	Wang et al., 2012
	Changing mitochondrial morphology, decreasing the activity of respiratory chain complex IV	Flies	Mortiboys et al., 2015
	Inducing mitochondrial disorder and dysregulation of autophagy	Primary neurons from rat midbrain, LRRK2 G2019S patient fibroblasts	Su et al., 2015
	Increasing inflammatory responses and apoptosis	Murine microglia	Kim et al., 2012
	Inducing dysfunctions in mitochondrial pathways, cell survival signaling, cancerization, endocytosis signaling and iron metabolism; deregulating the immune system and endocytosis	Peripheral blood mononuclear cell from patients	Mutez et al., 2014
R1441C	Inducing mitochondrial calcium dysregulation	Primary mouse cortical neurons	Verma et al., 2017
	Increasing mitochondrial DLP1, inducing mitochondrial fragmentation, and mitochondrial dysfunction	SH-SY5Y	Wang et al., 2012
	Inducing mitochondrial and lysosomal transport defects	SH-SY5Y	Thomas et al., 2016
I2020T and G2385R	Increasing LRRK2 GTPase Activity	HEK 293T	Ho et al., 2016


### PINK1 and Parkin

PTEN-induced putative kinase (PINK) 1, a mitochondrial protein kinase, is a critical protein linked with the pathogenesis of PD. PINK1 deficiency causes the autosomal recessive *PARK6* variant of PD. PINK1 mutations have been reported to account for approximately 1–3% of early onset PD in populations of European ancestry, 8.9% of autosomal recessive PD in a sample of Japanese families, and 2.5% of early onset PD in a sample of ethnic Chinese, Malays, and Indians (Bekris et al., 2010). PINK1 loss is associated with mitochondrial dysfunction and mitochondrial Ca^2+^ mishandling via directly interacting with and phosphorylating LETM1 at Thr192 site to increase Ca^2+^ release and facilitate its transport (Huang et al., 2017). In a *Caenorhabditis elegans* Model of PD, non-apoptotic neurodegeneration of dopaminergic cells is induced by the dysregulation of mitochondrial unfolded protein response, which is dependent on the PINK1 homolog (Martinez et al., 2017). Heterozygous PINK1 G411S mutation shows a significant decrease in kinase activity of PINK1 and could increase the risk of PD via a dominant-negative mechanism (Puschmann et al., 2017). Mutation of PINK1 show an accumulation of dysfunctional mitochondria with age, leading to activation of the mitochondrial unfolded protein response, which promotes longevity and dopamine neuron survival in PD models (Cooper et al., 2017). I368N and Q456X mutations of PINK1 reduce either its stability/protein levels or kinase activity, increasing the risk of PD (Ando et al., 2017). Other mutations, including A168P, H271Q, L347P and G309D, weaken the recruitment of Parkin to depolarized mitochondria (Narendra et al., 2010).

Parkin, encoded by *PARK2*, is originally considered to serve as a ubiquitin E3 ligase, which can be activated by autophosphorylated PINK1 (Zhuang et al., 2016) and is required for the ubiquitin-proteasome degradation of target substrates (Kumar et al., 2017). Mutations in the Parkin gene are a common cause of early onset PD in many countries and account for 10–25% of cases (Sanyal et al., 2015). PINK1 accumulates on the membrane of dysfunctional mitochondria, activates the E3 ubiquitin ligase activity of Parkin and recruits them to damaged mitochondria from cytoplasm to promote their mitophagic degradation (Narendra et al., 2009). Reactive oxygen species also induces PINK1/Parkin pathway-dependent mitophagy by promoting Parkin recruitment to mitochondria (Xiao et al., 2017). Mutations of Parkin, including R42P, R46P, K211N, C212Y, C253Y, C289G, and C441R, cause a deficit in its recruitment to depolarized mitochondria and further inhibit mitophagy (Narendra et al., 2010). Silence of PINK1/Parkin by siRNA promotes mitochondrial fragmentation in cultured primary neurons of mouse (Lutz et al., 2009). The mutations of PINK1 and Parkin linked to PD and the pathogenesis is summarized in **Table 3**.

**Table 3:** The mutations of PINK1 and Parkin linked to PD and the Pathogenesis.

Gene	Mutation	Pathogenesis	Cell type	Reference
PINK1	G411S	Reducing PINK1 kinase activity, impairing mitochondrial quality control	Neurons from PINK1 G411S carriers	Puschmann et al., 2017
	I368N	Reducing its stability and kinase activity	Fibroblasts	Ando et al., 2017
	Q456X	Reducing PINK1 protein levels and kinase activity	Neurons from PINK1 G411S carriers	Puschmann et al., 2017
	A168P, H271Q, L347P, and G309D	Inhibiting Parkin recruitment to depolarized mitochondria	MEF	Narendra et al., 2010
Parkin	R42P, R46P, K211N, C212Y, C253Y, C289G, and C441R	Causing a deficit in Parkin recruitment to depolarized mitochondria, inhibiting mitophagy	Hela	Narendra et al., 2010
	R275W	Inhibiting mitophagy	Hela	Narendra et al., 2010


### DJ-1

Mutations of DJ-1, encoded by the *PARK7* gene, have been linked with early onset of recessive PD, which were first identified in 2 European families with an age of onset of 20–40 years (Bonifati et al., 2003). Several PD-associated DJ-1 mutations have been identified, including L166P, M26I, L10P and P158Δ. Recently, genetic analysis from a single case research reveals a novel mutation of L172Q in the *PARK7* gene, which is the first time to report the neuropathology from a DJ-1-linked PD brain (Taipa et al., 2016). DJ-1 could interact with and steady PINK1 in cells overexpressing PINK1 (Tang et al., 2006). DJ-1 has been reported to also interact with α-synuclein, modulate its aggregation by weak hydrophobic interaction (Zhu et al., 2017) and restore α-synuclein-induced cellular toxicity (Zondler et al., 2014).

The neuroprotective roles mediated by DJ-1 are summarized as anti-oxidant properties, anti-apoptotic effects, effects on mitochondrial respiratory function, morphology, turnover and biogenesis (Mukherjee et al., 2015). Currently, the most acknowledged role of DJ-1 in the pathophysiology of PD is the neuroprotective function against oxidative stress (Biosa et al., 2017). DJ-1 mutations mainly affect a protein involved in intracellular oxidation-reduction (Ishikawa et al., 2009). Mutant DJ-1(L166P and M26I) increases the sensitivity of SHSY5Y cells toward oxidative stress, because it abolishes the neuroprotection against H_2_O_2_ and the induction of thioredoxin-1 through inhibiting nuclear factor erythroid-2 related factor 2 signal pathway (Im et al., 2012). D149A mutation also abolishes its protection (Giaime et al., 2010). The three mutations, L172Q, L10P and P158Δ, are involved in reduction of protein stability (Ramsey and Giasson, 2010; Taipa et al., 2016). DJ-1 deficiency in neurons shows a decrease of glutamine influx and serine biosynthesis, which attenuates cellular antioxidant response and leads to the degeneration of dopaminergic neurons (Meiser et al., 2016). DJ-1-Deficiecy in embryonic stem cells-derived dopaminergic neurons also increases the sensitivity to toxins-induced oxidative stress (Martinat et al., 2004). Drosophila carrying a mutant DJ-1β gene, the homologue of human DJ-1, show a reduced climbing ability reflecting locomotor defects (Sanz et al., 2017). DJ-1 overexpression ameliorates mitochondrial function, including the mitochondrial quantity and activity of respiratory chain complex I, by phosphorylating Akt at Thr308 site in both SH-SY5Y and PC-12 cells (Zhang et al., 2016). Recombinant DJ-1 can also reverse dopamine depletion in α-synuclein or 6-hydroxydopamine (6-OHDA)-triggered dopaminergic degeneration *in vivo* (Batelli et al., 2015). Some natural compounds protect against neurotoxin-induced Parkinsonism by upregulating DJ-1 expression. A marine compound exerts neuroprotective effects through upregulating DJ-1 expression and activating DJ-1-targeted pathway in 6-OHDA-induced zebrafish and rat models of PD (Feng et al., 2016). A standardized flavonoid extract from Safflower restores the expression of DJ-1, as well as the expression of tyrosine hydroxylase and dopamine transporter in rotenone-induced Parkinson rats (Ablat et al., 2016). The mutations of DJ-1 linked to PD and the pathogenesis is summarized in **Table 4**.

**Table 4:** The mutations of DJ-1 linked to PD and the Pathogenesis.

Mutation	Pathogenesis	Cell type	Reference
L166P	Dissociating Bax from mitochondrial Bcl-X_L_	HEK293 and H1299	Ren et al., 2012
	Shifting its subcellular distribution, decreasing its ability to dimerize and chaperone activity	SH-SY5Y and HEK293T	Deeg et al., 2010
	Abolishing its antioxidant activity	SH-SY5Y	Im et al., 2012
M26I	Abolishing its antioxidant activity	SH-SY5Y	Im et al., 2012
L10P and P158Δ	Reducing protein stability	CHO	Ramsey and Giasson, 2010
D149A	Abolishing its protection	HEK293 SH-SY5Y and MEF	Giaime et al., 2010
*DJ1*^-/-^	Increased sensitivity to oxidative stress	Embryonic stem cells	Martinat et al., 2004
L172Q	Reducing protein stability	HEK293	Taipa et al., 2016


### VPS35

Mutations of the Vacuolar protein sorting 35 (VPS35) gene were first discovered in an Austrian family with late-onset PD. It accounts for 1% of familial PD. VPS35, a major component of the retromer cargo-recognition complex, regulates the process of transmembrane protein sorting between endosomes and the Golgi (Bonifacino and Hurley, 2008). It has been reported that VPS35 D620N mutation is pathogenic in patients with PD in Asian, European and American families (Chen et al., 2017). VPS35 D620N mutation led to a decrease of enzymatic activity in complex I and II in PD patient fibroblasts (Zhou et al., 2017) and caused mitochondrial dysfunction by recycling DLP1 complexes (Wang W. et al., 2016). What’s more, VPS35 deficiency also led to mitochondrial dysfunction and finally resulted in the loss of dopaminergic neurons (Tang et al., 2015b). VPS35 mutant D620N disrupted the interaction of VPS35 with aminoacyl tRNA synthetase complex-interacting multifunctional protein 2 (AIMP2), a Parkin substrate, and lysosome-associated membrane protein-2a (LAMP2A). Overexpression of VPS35 inhibited AIMP2-enhanced cell death in SH-SY5Y cells; In contrast, downregulation of VPS35 resulted in AIMP2 dependent-cell death (Yun et al., 2017). VPS35-deficient DA neurons exhibit impaired endosome-to-Golgi retrieval of Lamp2a, which may contribute to the reduced α-synuclein degradation through chaperone-mediated autophagy (Tang et al., 2015a). Co-expression of high copy numbers of VPS35 D686N (the yeast equivalent of D620N) and low copy numbers of α-synuclein caused toxicity. Another PD-linked VPS35 mutation R524W impaired the endosomal association of retromer and also induced the aggregation of α-synuclein (Follett et al., 2016). In a Drosophila model, VPS35 P316S mutation also produced some of PD phenotypes, including reduction of climbing ability and loss of dopaminergic neurons, enhanced the susceptibility of Drosophila to rotenone (Wang et al., 2014). The mutations of VPS35 linked to PD and the pathogenesis is summarized in **Table 5**.

**Table 5:** The mutations of VPS35 linked to PD and the Pathogenesis.

Mutation	Pathogenesis	Cell type	Reference
D620N	Decreasing enzymatic activity and respiratory defects in complex I and II	PD patient fibroblasts	Zhou et al., 2017
	Causing mitochondrial dysfunction	Mouse SNc neurons and Primary fibroblast from a PD patient with D620N	Wang W. et al., 2016
	Disrupting the interaction of VPS35 with AIMP2 and LAMP2A; promoting cell death	SH-SY5Y	Yun et al., 2017
*VPS35*^-/-^	Impairing endosome-to-Golgi retrieval of Lamp2a and inhibiting autophagic degradation of α-synuclein	Midbrain primary dopaminergic neurons	Tang et al., 2015a
	Resulting in loss of dopaminergic neurons and accumulation of α-synuclein by impairing mitochondrial fusion and function	Mice and primary dopaminergic neurons	Tang et al., 2015b
R524W	Impairing the endosomal association of retromer and inducing α-synuclein aggregation	SH-SY5Y	Follett et al., 2016
P316S	Reducing the climbing abilities, loss of dopaminergic neurons	Drosophila	Wang et al., 2014


### GBA1

Glucocerebrosidase (GBA1) gene mutations, encoding gluco-cerebrosidase 1 (GCase 1) which catabolizes glycolipid glucocerebroside to ceramide and glucose in lysosome (Bae et al., 2015) and responsible for Gaucher disease, are the most common genetic risk factor for PD (Taguchi et al., 2017). It is recently reported that both heterozygous and homozygous mutations of the GBA1 gene have been linked to PD, with more than 10% of individuals carrying GBA1 mutations developing PD by the age of 80 (Migdalska-Richards et al., 2016). A significant and progressive reduction in GCase 1 activity and protein levels is also observed in the brains and cerebrospinal fluid of PD patients (Murphy et al., 2014; Rocha et al., 2015; Parnetti et al., 2017). Activation of GCase 1 by using a small-molecule modulator restored the function of lysosome and then cleared the accumulation of pathological α-synuclein in midbrain neurons of PD patients (Mazzulli et al., 2016), indicating the potential importance of GCase 1 in the development of idiopathic PD. It is estimated that about 10–25% of PD cases carry a GBA1 mutation, in which N370S and L444P are the most common mutations (Sidransky et al., 2009; McNeill et al., 2012). GBA1 knockout inhibited macroautophagy and chaperone-mediated autophagy in MEFs and downregulation of GCase 1 increased α-synuclein protein levels in SH-SY5Y cells (Magalhaes et al., 2016). GBA1 deficiency also resulted in the accumulation of glycosphingolipids (GSL), then decreased α-synuclein tetramers resisting aggregation and related multimers and increased α-synuclein monomers in CRISPR-GBA1 knockout SH-SY5Y cells. What’s more, α-synuclein tetramers and related multimers were also reduced in PD induced pluripotent stem cell (iPSC)-derived human dopaminergic neurons carrying N370S GBA1 mutation (Kim et al., 2018). In contrast, inhibition of GSL accumulation and overexpression of GBA1 to enhance GCase 1 activity reverse the destabilization of α-synuclein tetramers and protect against α-synuclein preformed fibril-induced toxicity in human dopaminergic neurons. N370S mutation produced GCase 1 retention within the endoplasmic reticulum, interrupting its traffic to the lysosome, leading to endoplasmic reticulum stress activation and triggering unfolded protein response and Golgi apparatus fragmentation (Garcia-Sanz et al., 2017, 2018). Decreased α-synuclein tetramers and related multimers were also observed in murine neurons carrying the heterozygous L444P GBA1 mutation (Kim et al., 2018) and L444P GBA1 mutation aggravated α-synuclein-induced loss of dopaminergic neurons in SNc of 24-month-old mice (Migdalska-Richards et al., 2017). The mutations of GBA1 linked to PD and the pathogenesis is summarized in **Table 6**.

**Table 6:** The mutations of GBA1 linked to PD and the Pathogenesis.

Mutation	Pathogenesis	Cell type	Reference
*GBA1*^-/-^	inhibiting macroautophagy and chaperone-mediated autophagy	MEF	Magalhaes et al., 2016
	Resulting in the accumulation of GSLs, decreasing α-synuclein tetramers and related multimers, increasing α-synuclein monomers	SH-SY5Y	Kim et al., 2018
N370S (heterozygous)	Decreasing α-synuclein tetramers and related multimers	PD iPSC-derived human dopaminergic neurons	Kim et al., 2018
	Leading to endoplasmic reticulum stress activation and triggering unfolded protein response and Golgi apparatus fragmentation	Fibroblasts from skin biopsies of PD patients	Garcia-Sanz et al., 2017
	Reducing lysosomal Ca^2+^ store content	PD patient fibroblasts	Kilpatrick et al., 2016
L444P (heterozygous)	Decreasing α-synuclein tetramers and related multimers	Murine neurons carrying heterozygous L444P GBA1 mutation	Kim et al., 2018
	Aggravating α-synuclein-induced loss of nigral dopaminergic neurons	Mice	Migdalska-Richards et al., 2017


## Mechanisms of Neuronal Cell Death in PD

The most obvious progress has been made in understanding the proximate causes, especially the deaths of dopaminergic neurons that explain the classic motor symptoms of the disease. The deaths mainly occur in the SNc, in which the neurotransmitter dopamine is normally synthesized and pumped into movement-regulating brain regions. Though up to 70% of these dopaminergic neurons in SNc will be killed during the development of PD, symptoms are seldom noticed because the striatum, immediately downstream of the SNc, can compensate to some extent. However, massive neurons in the striatum also start to die eventually. Currently, it is widely accepted that dopamine metabolism, oxidative stress and mitochondrial dysfunction, endoplasmic reticulum stress, impairment of protein degradation systems, and neuroinflammation are involved in the death of dopaminergic neurons in SNc (**Table 7**).

**Table 7:** Main mechanisms involving the death of the dopaminergic neuron.

Element	Main mechanism
Dopamine	Dopamine oxidation induces oxidative stress
Mitochondria	Inhibition of respiratory chain induces oxidative stress
Proteins aggregation	Unfolded proteins induce severe endoplasmic reticulum stress-mediated neuronal apoptosis
Autophagy	Impaired autophagy fails to degrade accumulated misfolded proteins and damaged organelles
Neuroinflammation	Microglial activation induces the neurodegeneration
Calcium	Reliance on Ca^2+^ channels for pacemaking accelerates the aging and loss of SNc dopaminergic neurons.


### Dopamine Metabolism

Parkinson disease is a movement disorder and the first neurodegenerative disease, which is confirmed severe depletion of dopamine (regulates Parkinson’s tremor) in the striatum (Dirkx et al., 2017). Oxidation of dopamine could induce oxidative stress and is harmful to dopaminergic neurons. Mitochondrial oxidative stress results in the accumulation of oxidized dopamine and ultimately leads to reduced activity of glucocerebrosidase, lysosomal dysfunction, and α-synuclein accumulation in human and PD neurons (Burbulla et al., 2017; Garcia-Sanz et al., 2017). Increased levels of dopamine in cytoplasm and its metabolites are neurotoxic and lead to selective death of neurons in SNc, administration that decreases cytoplasmic dopamine levels provide neuroprotection in cultured midbrain neurons (Mosharov et al., 2009). Aminochrome, which is formed within the cytoplasm during the auto-oxidation of dopamine to neuromelanin, could induce neurotoxicity in dopaminergic neurons when DT-diaphorase and glutathione transferase mutate (Herrera et al., 2017). Auto-oxidation of dopamine aggravates overexpression of human α-synuclein A53T mutation-induced non-apoptotic cell death in PC12 cells (Zhou et al., 2009). Dopamine not only spontaneously oxidize to form melanin and neuromelanin, but also is catalyzed to the immediate products being hydrogen peroxide and aldehydes by monoamine oxidase (Goldstein et al., 2014). Monoamine oxidase in the outer membrane of mitochondria catalyzes conversion of cytosolic dopamine to 3,4-dihydroxyphenylacetaldehyde, which is an endogenous neurotoxin implicated in PD (Panneton et al., 2010), cause extensive aggregation of glyceraldehyde-3-phosphate dehydrogenase and irreversibly inhibit the enzyme activity, resulting in the production of reactive oxygen species to damage neurons (Vanle et al., 2017). Dopamine also induces the production of soluble A53T α-synuclein oligomers and promotes the degeneration of neurons in SNc (Mor et al., 2017). Vesicular uptake inhibition of dopamine with Reserpine rapidly increases the cytosolic concentration of 3,4-dihydroxyphenylacetaldehyde, contributing to the dimerization of α-synuclein and apoptosis (Goldstein et al., 2012). Dopamine induces mitochondrial permeability transition pore opening via production of reactive oxygen species in mouse neurons with PINK1 defect (Gandhi et al., 2012). Reuptake of extracellular dopamine to cytoplasm leads to accumulation of dopamine, which triggers neurotoxicity in dopaminergic neurons. Disrupting the interaction between dopamine D2 receptor and dopamine transporter provides a protective role against dopamine neurotoxicity (Su and Liu, 2017).

### Mitochondrial Dysfunction

Mitochondrial disorders have been implicated in almost all neurodegenerative diseases, including PD (Subramaniam and Chesselet, 2013; Connolly et al., 2017). PD-linked mutations could induce mitochondrial dysfunction. Soluble prefibrillar α-synuclein oligomers showed several phenotypes of mitochondrial dysfunction observed previously in cell and animal models of PD: dysfunction of complex I, change in membrane potential, disruption of Ca^2+^ homeostasis, and enhanced release of cytochrome c (Luth et al., 2014). PD-associated mutation of *VPS35* gene, encoding a key component of the retromer complex, leaded to mitochondrial fragmentation and neuronal death *in vitro* and *in vivo* by enhanced interactions with DLP1 (Wang W. et al., 2016). Inhibiting the enzymatic activity of the GCase 1, or silencing the GBA1 gene in SH-SY5Y, also caused mitochondrial dysfunction, showing reduced activity of mitochondrial respiratory chain and mitochondrial depolarization and fragmentation associated with increased levels of ROS (Cleeter et al., 2013). In the SNc of idiopathic PD patients, the expression of mitochondrial DNA transcription factors (TFAM and TFB2M) is decreased. Consistent with this, real-time PCR analysis exhibits a reduced copy number of mitochondrial DNA and fewer molecules associated with transcription/replication in PD patients (Grunewald et al., 2016). 4-hydroxynonenal, a lipid peroxidation product, promotes the intracellular accumulation, extrusion of extracellular vesicles containing toxic α-synuclein, and then internalization into neighboring neurons, finally results in the neurodegenerative development of PD (Zhang et al., 2018).

Numerous studies have repeatedly demonstrated that the expression and activity of respiratory chain complex I are markedly reduced in PD (Yin et al., 2014). The initial evidence is that exposure to environmental 1-methyl-4-phenyl-1,2,3,6-tetrahydropyridine (MPTP) caused degeneration of dopaminergic neurons and parkinsonism (Langston et al., 1983). MPTP, a PD neurotoxin, is metabolized to its active metabolite MPP^+^ by monoamine oxidase and then is transferred into dopaminergic neurons by dopamine transporter (Storch et al., 2004). MPP^+^ is considered as an inhibitor of mitochondrial complex I, inhibits ATP production, then results in the generation of superoxide and peroxynitrite, and finally ablates dopaminergic neurons (Khaldy et al., 2003; Yan et al., 2013). Partial deficiency of mitochondrial complex I in dopaminergic neurons increases the vulnerability of mice toward MPTP (Sterky et al., 2012). Other neurotoxins can also induce degeneration of dopaminergic neurons and parkinsonism. The herbicide paraquat, reduced by NADPH oxidase on microglia, is also transported into dopaminergic neurons by dopamine transporter, where it induces mitochondrial dysfunction and neurotoxicity (Rappold et al., 2011). Injection of PD neurotoxin 6-OHDA into the right SNc also reduces activity of mitochondrial oxidative phosphorylation enzymes, particularly selective decrease of complex I activity (Dabbeni-Sala et al., 2001). Chronic treatment of rodents with pesticide rotenone, a well-established complex I inhibitor, selectively induces toxicity to dopaminergic neurons (Betarbet et al., 2000; Choi et al., 2011).

There is a potential interaction between environmental neurotoxin-induced mitochondrial dysfunction and genetic risk factors in PD pathogenesis. Injection of sub-toxic MPTP enhances the neurotoxicity of mutant α-synuclein and induces vulnerability of dopamine neurons, which may be due to the downregulation of DJ-1 (Lee et al., 2017). The higher level of neurotoxicity in SNc than VTA neurons is due to SNc neuron-specific MPP^+^-induced increase in cytosolic dopamine and Ca^2+^, followed by an elevation of mitochondrial Ca^2+^ and mitochondrial oxidation, which is dependent on the expression of α-synuclein (Lieberman et al., 2017). A long non-coding RNA promotes MPTP-induced parkinsonism via upregulating the expression of LRRK2 (Liu et al., 2016). Mice expressing LRRK2 G2019S mutation exhibit elevated susceptibility to MPTP-induced neurotoxicity (Karuppagounder et al., 2016). Downregulation of PINK1 enhances the sensitivity of dopaminergic neurons to MPTP-induced neurotoxicity, which can be inhibited by overexpression of Parkin or DJ-1 (Haque et al., 2012).

### Endoplasmic Reticulum Stress

Parkinson disease is a disease classified as protein-misfolding disorders. Endoplasmic reticulum stress is observed in the SNc of PD patient brain and results in the death of dopaminergic neurons (Selvaraj et al., 2012). Increasing experimental evidence have also indicated that the accumulation of misfolded or unfolded proteins results in endoplasmic reticulum stress, which contributes to the neuronal death or apoptosis (Ryu et al., 2002; Bai et al., 2007; Colla et al., 2012; Fouillet et al., 2012; Valdes et al., 2014). In the central nervous system, the unfolded protein response(UPR) is initiated by the activation of three transmembrane proteins: (1) PRKR-like ER kinase (PERK), (2) inositol-requiring enzyme 1α (IRE1α) and (3) activating transcription factor 6 (ATF6) (Kaufman, 2002; Schroder and Kaufman, 2005). Under stressed conditions, the three transmembrane proteins dissociate from glucose regulated protein 78(GRP78) and activate series of intracellular signals to alleviate the burden of endoplasmic reticulum, such as inhibiting transcription and translation to reduce the amount of proteins entering the endoplasmic reticulum, increasing the levels of molecular chaperones to enhance the folding capacity of endoplasmic reticulum (Walter and Ron, 2011).

Mild endoplasmic reticulum stress is considered to have a neuroprotective role. However, severe endoplasmic reticulum stress usually leads to apoptosis or death of neurons. IRE1α/XBP1 is the most conserved UPR signaling pathway and a key regulator of UPR. IRE1α/XBP1 signal pathway of UPR is activated in *SNCA* triplication iPSC neurons, resulting in culminating neurodegeneration (Heman-Ackah et al., 2017). Conditional knockout of XBP1 in the nervous system inhibits the death of dopaminergic neurons induced by stereotaxic injections of 6-OHDA (Valdes et al., 2014). Our previous study also demonstrates that MPP^+^/MPTP activates IRE1α pathway, leading to the loss of dopaminergic neurons *in vitro* and *in vivo*; Thioredoxin-1 exerts a neuroprotective effect via inhibiting the activation of IRE1α (Zeng et al., 2014). Overexpression of human α-synuclein activates endoplasmic reticulum stress via activation of PERK and ATF6 signaling pathways as well as the upregulation of proaoptotic CHOP in dopaminergic neurons in SNc, which is inhibited by overexpression of glucose regulated protein 78 (Gorbatyuk et al., 2012). Rifampicin protects PC12 cells against the cytotoxicity of PD neurotoxin rotenone by upregulating GRP78 expression via PERK/eIF2α/ATF4 pathway (Jing et al., 2014). Endoplasmic reticulum stress signaling is enhanced in PINK1 and Parkin mutants model of PD, and genetic or pharmacological inhibition of PERK is protective (Celardo et al., 2016). The N370S mutation of GBA1 gene produces a significant reduction in GCase 1 protein and enzyme activity, and retention within the endoplasmic reticulum, which interrupted its traffic to the lysosome and further led to endoplasmic reticulum stress activation and triggered UPR and Golgi apparatus fragmentation(Garcia-Sanz et al., 2017). Sustained activation of ATF4 does not repress human alpha-synuclein overexpression-induced neurodegeneration and induces severe apoptosis of dopaminergic neurons in a rat model of PD (Gully et al., 2016). Recently,Coppola-Segovia et al. (2017) have presented a new model of PD, intranigral injection of Tunicamycin, in which the endoplasmic reticulum stressor recapitulates some of the phenotypic characteristics observed in rodent models of PD (such as locomotor impairment, α-synuclein oligomerization, and loss of dopaminergic neurons), reinforcing that endoplasmic reticulum stress could be an important contributor to the development of PD (Coppola-Segovia et al., 2017).

### Impaired Autophagy

Autophagy plays a critical role in the clearance of accumulated misfolded proteins and degradation of damaged organelles. Resembling endoplasmic reticulum stress, the initial autophagy is also widely considered to be cyto-protective. Overexpression of autophagy-related gene 5 inhibits the apoptosis of dopaminergic neurons in MPTP-induced zebrafish model of PD (Hu et al., 2017). It can be achieved to inhibit the accumulation of α-synuclein by activating mTOR-dependent macroautophagy (Sheng et al., 2017). DJ-1 reduced the accumulation and aggregation of α-synuclein via chaperone-mediated autophagy in both SH-SY5Y cells and PD animal models (Xu et al., 2017). Interaction between PINK-1 and α-synuclein in the cytoplasm, which is dependent on the kinase activity of PINK1 and is abolished by point mutation of G309D in its kinase domain, enhanced the degradation of toxic α-synuclein via activation of autophagy (Liu et al., 2017). Endoplasmic reticulum stressors, including thapsigargin, tunicamycin, 2-mercaptoethanol and Brefeldin A, activate PERK and then recruit MKK4 to lysosomes, accompanied by the activation of p38 MAPK which directly phosphorylates the receptor LAMP2A at sites T211 and T213, and further triggers chaperone-mediated autophagy to provide a pro-survival mechanism (Li et al., 2017).

However, autophagy is usually defective or inhibited in the pathology of PD. Impairment of autophagy has been implicated as a causative mechanism in the development of PD (Cai et al., 2016). The autophagy levels in dopaminergic brain areas of α-synuclein mutant A53T transgenic mice are reduced when compared to wide-type mice (Pupyshev et al., 2017). Inhibition of autophagy boosts α-synuclein release and transfer from cell to cell via extracellular vesicles, and consequent progression of synucleinopathies (Minakaki et al., 2017). Cuervo et al. (2004) have demonstrated that pathogenic α-synuclein mutations, such as A53T and A30P, could bind to the receptors on the lysosomal membrane and inhibit chaperone-mediated autophagy, resulting in accumulation of abnormal α-synuclein in the Lewy bodies and subsequent parkinsonism. Inhibition of chaperone-mediated autophagy results in progressive loss of dopaminergic neurons in SNc, severe decrease of dopamine levels in striatum, and motor deficits (Xilouri et al., 2016). The mitochondria of neurons, suffering from oxidative stress and dysfunction, can’t be removed by impaired mitophagy in PD, leading to the accumulation of dysfunctional mitochondria (Gao et al., 2017). The formation of S-Nitrosylated PINK1 reduces the translocation of Parkin to mitochondrial membranes and inhibits mitophagy in human iPSC-derived cells as well as in α-synuclein transgenic PD mice, which contributes to neuronal cell death (Oh et al., 2017). GBA1 mutation produces an accumulation of cholesterol, which alters autophagy-lysosome function and impairs autophagy, rendering the neurons more vulnerable and sensitive to apoptosis(Garcia-Sanz et al., 2017, 2018). Elevated RTP801, product of *DNA damage inducible transcript 4*, represses autophagy, then increases the accumulation of oligomeric α-synuclein and further aggravates ER stress-induced neuronal apoptosis (Zhang et al., 2017).

### Deregulation of Immunity

Increasing evidence indicates that the immune system plays a role in the development of PD. Several previous studies have demonstrated that pro-inflammatory cytokines, such as TNF-α, IL-1β, IL-6 and IFN-γ, are upregulated in both cerebrospinal fluid and post-mortem brain of PD patients (Mogi et al., 1994a,b; Blum-Degen et al., 1995; Brodacki et al., 2008). Massive studies have demonstrated that microglia are activated in neurotoxins-induced PD models, including MPTP, 6-OHDA and rotenone (Akiyama and McGeer, 1989; McGeer et al., 2003; Sherer et al., 2003).

There is an intimate relation between α-synuclein and immune system (Bandres-Ciga and Cookson, 2017). In a rat α-synuclein overexpressing model of PD, microglia are also activated in the striatum, accompanying the upregulation of TNF-α, IL-1β, and IFN-γ (Chung et al., 2009). Lack of IFN-β function shows an increase of α-synuclein-containing Lewy bodies, a reduction of dopaminergic neurons and impairment of dopamine signaling in SNc. In contrast, overexpression of IFN-β inhibits the loss of dopaminergic neuron in a model of familial PD (Ejlerskov et al., 2015). A recent study has showed that T cells from PD patients recognize α-synuclein peptides as antigenic epitopes which may explain the association of PD with specific major histocompatibility complex alleles (Sulzer et al., 2017). Major histocompatibility complex I is expressed in human SNc neurons and is inducible in human stem cell-derived dopaminergic neurons. Dopaminergic neurons internalize foreign ovalbumin and display antigen derived from this protein by major histocompatibility complex I to activate T cells, resulting in autoimmune responses and dopaminergic neurons death (Cebrian et al., 2014). α-synuclein induces the entry of pro-inflammatory peripheral CCR^2+^ monocytes into SNc, promotes the expression of major histocompatibility complex II and the subsequent degeneration of dopaminergic neurons (Harms et al., 2018). Genetic allelic variants of Mhc2ta, the major regulator of major histocompatibility complex II expression, regulates α-synuclein-induced microglial activation and the neurodegeneration of dopaminergic neurons (Jimenez-Ferrer et al., 2017).

Other PD-linked gene mutations also exhibit an association with immune system. Immune system and endocytosis are involved in the pathology of sporadic PD forms and LRRK2 G2019S carrying patients (Mutez et al., 2014). LRRK2 G2019S could alter the differentiation of bone marrow myelopoiesis and immune homeostasis (Park et al., 2017). PINK1 deficiency serves as an early modulator of innate immunity in neurons(Torres-Odio et al., 2017), because PINK1/Parkin could suppress inflammatory conditions triggered immune-response-eliciting pathway by inhibiting mitochondrial antigen presentation (Matheoud et al., 2016). At last, we may draw a conclusion that PD is an autoimmune disorder and deregulation of immunity is responsible for parkinsonism.

## Conclusion and Expectation

In summary, pathology of PD is compositive and due to the combined action of heredity and environmental factors. Mutations of single-gene or multi-genes could aggravate the effects of environmental neurotoxins. The pathogenesis of mutations linked to PD are associated with dysfunctions of mitochondria and endoplasmic reticulum, impairment of autophagy, and deregulation of immunity. Conversely, environmental neurotoxins could also deregulate the expression of PD-linked genes.

Although PD is widely researched and the therapy is positively explored, it is still incurable and persecutes tens of million sufferers worldwide. Many scientists are unremittingly searching for potential treating approaches. Mao et al. (2016) have demonstrated that lymphocyte-activation gene 3 interact with misfolded preformed fibrils of α-synuclein, initiates its neuron-to-neuron transmission and then induces loss of dopaminergic neurons (Mao et al., 2016). Using an unbiased screen targeting endogenous gene expression, Mittal et al. (2017) have discovered that the β_2_-adrenoreceptor (β_2_AR) is a regulator of the α-synuclein gene. Salbutamol, a β_2_AR agonist, reduced the risk of developing PD. On the other hand, a β_2_AR antagonist correlated with increased risk. What’s more, activation of β_2_AR protected PD patient-derived cells and model mice (Mittal et al., 2017). Gut bacteria are required for motor deficits, microglia activation, and α-synuclein pathology, suggesting that alterations in the human microbiome may increase the risk for PD (Sampson et al., 2016). Targeting these aspects may provide effective and safe treatments to halt or slow the progression of PD.

Shields et al. (2017) have developed a DART (drugs acutely restricted by tethering), which antagonizes the a-amino-3-hydroxy-5-methylisoxazole-4-propionic acid receptor (AMPAR, a postsynaptic glutamate receptor), and applied the technique to a mouse model of PD. Human induced pluripotent stem cells-derived dopaminergic progenitor cells could function as midbrain dopaminergic neurons after transplantation in a monkey model of PD (Kikuchi et al., 2017). Using three transcription factors (NEUROD1, LMX1A, and ASCL1), miR218 and collectively designated NeAL218, dopamine neurons are generated by directly reprogramming human astrocytes *in vitro* and mouse astrocytes *in vivo* (Rivetti di Val Cervo et al., 2017). Ambroxol, which was initially used to treat airway mucus hypersecretion and hyaline membrane disease in infants and was then recognized as a pH-dependent, mixed-type inhibitor of GCase1, acts as a chaperone for GCase1 and enhances lysosomal function and autophagy. Clinical trials of ambroxol are currently ongoing in PD and PD dementia (Balestrino and Schapira, 2018). These studies might also provide new and alternative strategies to treat PD.

## Author Contributions

X-SZ and J-JJ designed the article contents and wrote the original paper. X-SZ, W-SG, J-JJ, LC, and P-PZ searched the related literature and discussed the writing. X-SZ, W-SG, and J-JJ revised the paper.

## Conflict of Interest Statement

The authors declare that the research was conducted in the absence of any commercial or financial relationships that could be construed as a potential conflict of interest.
